# Supplementary material for: Evidence supported by Mendelian randomization: impact on inflammatory factors in knee osteoarthritis
Source: Front Med (Lausanne). 2024 May 28;11:1382836. doi: 10.3389/fmed.2024.1382836 (PMC11165061; doi:10.3389/fmed.2024.1382836)
Supplement: Supplementary file 1 [file Data_Sheet_1.ZIP › data/GCST90274771_5e6/771.forest.pdf]

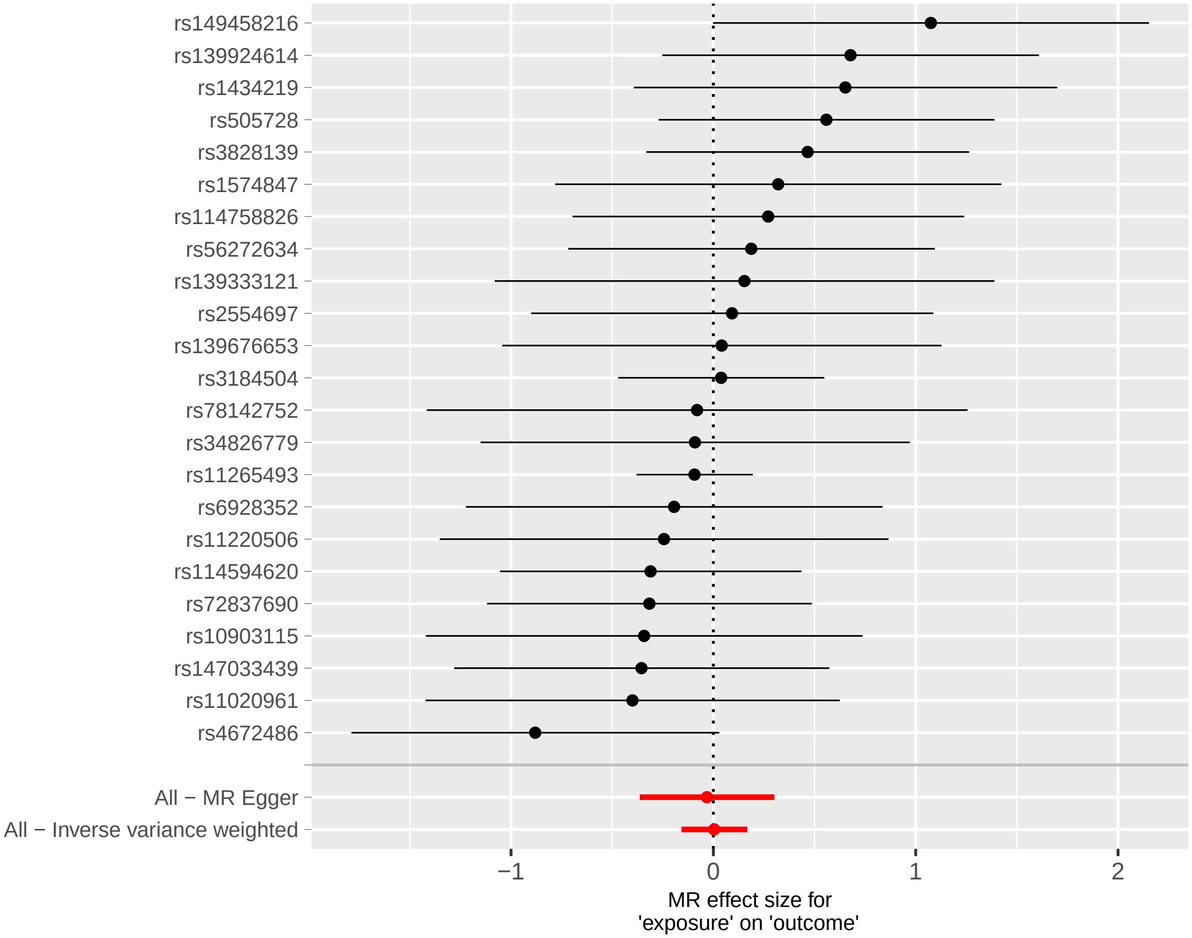
rs149458216
rs139924614
rs1434219
rs505728
rs3828139
rs1574847
rs114758826
rs56272634
rs139333121
rs2554697
rs139676653
rs3184504
rs78142752
rs34826779
rs11265493
rs6928352
rs11220506
rs114594620
rs72837690
rs10903115
rs147033439
rs11020961
rs4672486
All − MR Egger
All − Inverse variance weighted
−1
0
1
2
MR effect size for
'exposure' on 'outcome'
